# Cardiovascular magnetic resonance findings in young adult patients with acute myocarditis following mRNA COVID-19 vaccination: a case series

**DOI:** 10.1186/s12968-021-00795-4

**Published:** 2021-09-09

**Authors:** Yash R. Patel, David W. Louis, Michael Atalay, Saurabh Agarwal, Nishant R. Shah

**Affiliations:** 1 Division of Cardiology, Department of Medicine, Warren Alpert Medical School of Brown University, Providence, RI USA; 2 Department of Diagnostic Imaging, Warren Alpert Medical School of Brown University, Providence, RI USA; 3 Lifespan Cardiovascular Institute, Warren Alpert Medical School at Brown University, 950 Warren Avenue, Suite 201, East Providence, RI 02914 USA

**Keywords:** COVID-19 infection, Acute myocarditis, Acute myopericarditis, COVID-19 vaccination

## Abstract

**Background:**

Messenger RNA (mRNA) coronavirus disease of 2019 (COVID-19) vaccine are known to cause minor side effects at the injection site and mild global systemic symptoms in first 24–48 h. Recently published case series have reported a possible association between acute myocarditis and COVID-19 vaccination, predominantly in young males.

**Methods:**

We report a case series of 5 young male patients with cardiovascular magnetic resonance (CMR)-confirmed acute myocarditis within 72 h after receiving a dose of an mRNA-based COVID-19 vaccine.

**Results:**

Our case series suggests that myocarditis in this setting is characterized by myocardial edema and late gadolinium enhancement in the lateral wall of the left ventricular (LV) myocardium, reduced global LV longitudinal strain, and preserved LV ejection fraction. All patients in our series remained clinically stable during a relatively short inpatient hospital stay.

**Conclusions:**

In conjunction with other recently published case series and national vaccine safety surveillance data, this case series suggests a possible association between acute myocarditis and COVID-19 vaccination in young males and highlights a potential pattern in accompanying CMR abnormalities.

## Background

Coronavirus disease of 2019 (COVID-19), caused by severe acute respiratory syndrome coronavirus 2 (SARS-CoV-2), has affected 169 million people across the globe and has resulted in more than 3.5 million deaths [1]. Two messenger RNA (mRNA) vaccines (Pfizer-BioNTech, Moderna) are now United States (US) Food and Drug Administration (FDA)-approved to reduce the risk and severity of COVID-19 infection [2]. To date, 294.9 million COVID-19 vaccination doses have been administered in the US [3] and the FDA recently expanded emergency use authorization of the Pfizer COVID-19 vaccine to include minors 12 years and older [2]. On May 17, 2021, the US Centers for Disease Control and Prevention (CDC) reported several cases of myocarditis within four days after receiving an mRNA-based COVID-19 vaccine, particularly in younger males after the second vaccine dose [4]. A handful of subsequently published case series also suggests a possible association between acute myocarditis and mRNA-based COVID-19 vaccination in young adult males [5–10] and in pediatric patients [11]. Based on the evidence currently available, the CDC continues to recommend COVID-19 vaccination for everyone 12 years or older [12]. In this case series, we report the patient characteristics, cardiovascular magnetic resonance (CMR) findings, and clinical course of 5 young male patients with acute myocarditis within 72 h after mRNA-based COVID-19 vaccination.

## Methods

### Case series

This case series includes 5 young males who were diagnosed with myocarditis or myopericarditis within 72 h after receiving a dose of either the Pfizer or Moderna mRNA-based COVID-19 vaccine.

### Results

**Fig. 1:**
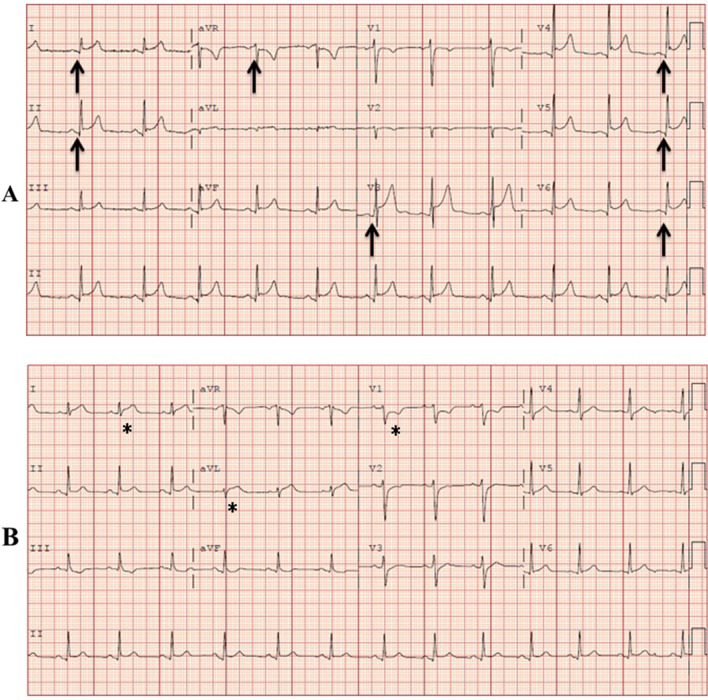
ECG findings. **A** 12-lead electrocardiogram (ECG) from Case 1 showing diffuse PR segment depression and PR segment elevation in lead aVR (arrows). Similar ECG findings were present in Cases 3 and 5. **B** 12-Lead ECG from Case 4 showing ST segment elevation in lateral leads and ST segment depression in lead V1 (asterisks)

**Fig. 2:**
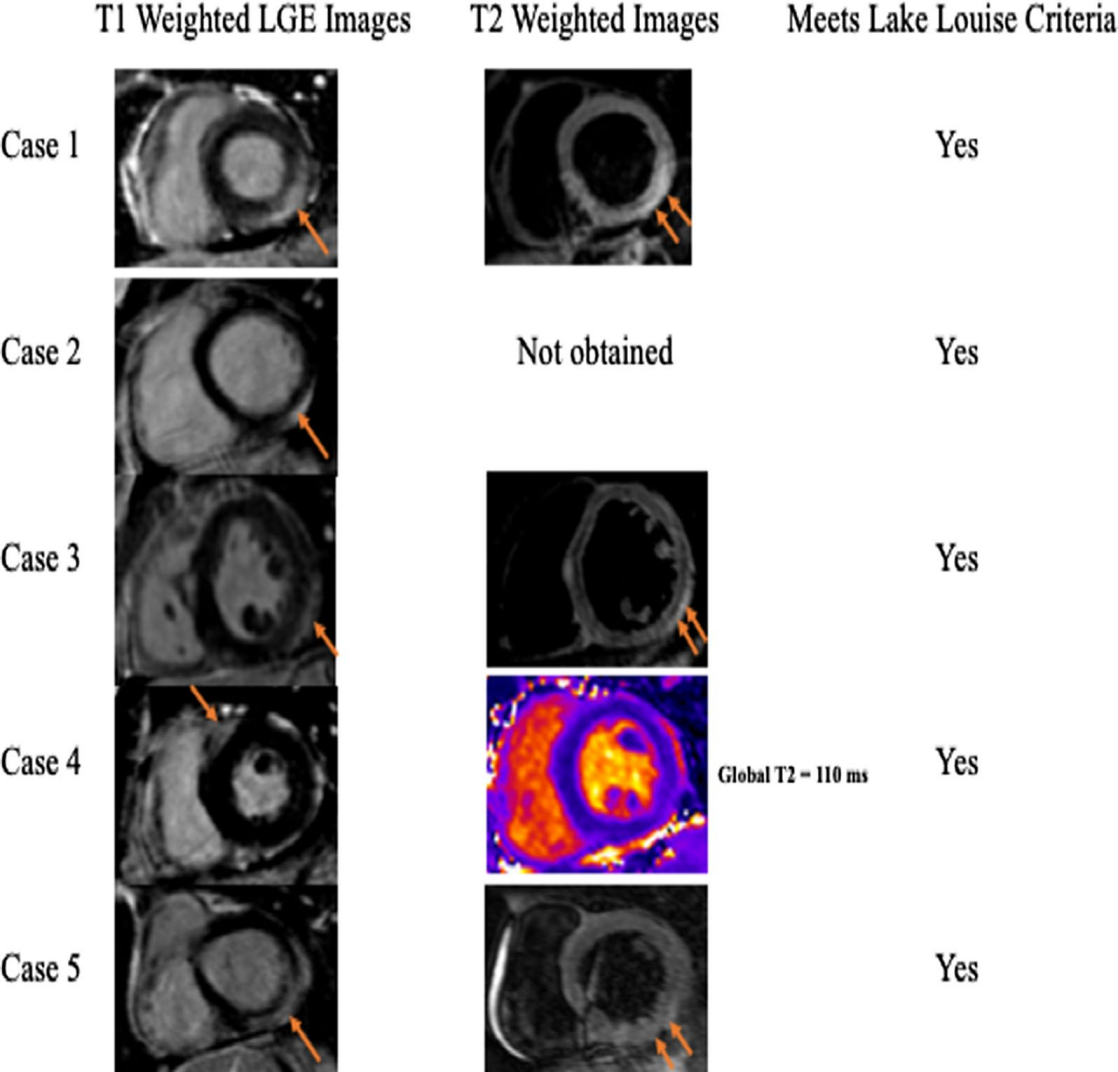
Cardiovascular magnetic resonance (CMR) findings. Late gadolinium enhancement on T1-weighted images (single arrow) and hyperintense signal suggestive of myocardial edema on T2-weighted fat suppressed images (double arrow). In Case 4, there was no hyperintense signal on T2-weighted images but global T2 mapping showed an elevated relaxation time of 110 ms (normal range < 60 ms), consistent with myocardial edema. CMR findings met updated 2018 Lake Louise criteria for acute myocarditis [14] for all cases

Key characteristics of each case are described below, with additional relevant clinical information included in Table 1. Representative 12-lead electrocardiogram (ECG) findings are shown in Fig. 1 and key CMR findings from each case are shown in Fig. 2.

**Table 1:** Clinical characteristics of patients with acute myocarditis/myopericarditis following COVID-19 vaccination

	Case 1	Case 2	Case 3	Case 4	Case 5
Age (years)	22	19	25	37	20
Gender	Male	Male	Male	Male	Male
Body mass index (kg/m^2^)	24	25	28	28	26
Coronary artery disease risk factors	No	No	No	No	No
Cardiac comorbidities	No	No	No	No	No
Symptoms					
Chest pain	Yes	Yes	Yes	Yes	Yes
Dyspnea	No	Yes	Yes	No	Yes
Other	Headache, generalized malaise	Nausea, emesis	Generalized body aches, nausea, headache, chills, and fatigue	Fever, diaphoresis, rigors, nausea, myalgia, headache, fatigue	Headache, body ache
Arrhythmia	No	No	No	No	No
COVID-19 vaccine doses prior to symptom onset	1	2	2	2	2
COVID-19 vaccine manufacturer	Pfizer	Pfizer	Moderna	Pfizer	Pfizer
Days after vaccine administration prior to symptom onset	2	1	3	2	3
Cardiac rub	No	No	No	No	No
Serum TnI (ng/ml)	37	49	17	26	58
Brain natriuretic peptide (pg/ml)	Not obtained	Not obtained	Not obtained	Not obtained	133
Erythrocyte sedimentation rate (mm/hr)	Not obtained	26	24	32	20
C-reactive protein (mg/l)	50	109	96	Not obtained	Not obtained
SARS COVID-19 PCR testing	Negative	Negative	Negative	Negative	Negative
Other Viral Serologies					
Adenovirus	Not detected	Not detected	Not detected	Not obtained	Not detected
Coxsackie antibody	Not obtained	Not detected	Not obtained	Not obtained	Negative
Echovirus Titer	Not obtained		Not obtained	Not obtained	Negative
Rhinovirus	Not detected	Not detected	Not detected	Not obtained	Not detected
Influenza/ Parainfluenza	Not detected	Not detected	Not detected	Not detected	Not detected
Parvovirus B19	Not obtained	Not detected	Not obtained	Not obtained	Not detected
Respiratory Syncytial Virus	Not detected	Not detected	Not detected	Not detected	Not detected
Epstein-Barr Virus	Not obtained	Not detected	Not obtained	Not obtained	Not obtained
Bacterial test					
Lyme antibodies	Negative	Not detected	Not obtained	Negative	Negative
Mycoplasma pneumoniae	Not detected	Not detected	Not detected	Not obtained	Not detected
Chlamydia pneumoniae	Not detected	Not detected	Not detected	Not obtained	Not detected
TTE LVEF (%)	55	62	60	65	51
CMR LVEF (%)	65	59	59	61	59
TTE Global longitudinal strain (%)	− 19.0	− 12.0	− 21.0	− 18.0	− 14.0
CMR Global longitudinal strain (%)	− 19.1	− 12.1	− 19.6	− 16.0	− 12.2
Pericardial effusion	No	No	No	No	No
Anatomic coronary artery assessment	Non-obstructive (ICA)	Not obtained	Not obtained	Non-obstructive (ICA)	Non-obstructive (CCTA)
Oxygen support	No	No	No	No	No
Length of stay (days)	2	2	1	2	2
Death	No	No	No	No	No
Discharge medications	High-dose aspirin, colchicine	Colchicine, high-dose ibuprofen	Colchicine	None	Colchicine, high-dose ibuprofen, lisinopril, metoprolol tartrate

*CCTA* coronary CT angiography, *COVID-19* the coronavirus disease of 2019, *ICA* invasive coronary angiography, *LVEF* left ventricular ejection fraction, *CMR* cardiovascular magnetic resonance*Serum C-reactive protein level (normal range 0–10 mg/l); serum erythrocyte sedimentation rate (normal range 0–15 mm/h); serum troponin I level (normal range 0.006–0.060 ng/ml); normal global longitudinal strain is less than negative 18%

#### Case 1

A 22-year-old male with a history of attention deficit hyperactive disorder and without any known cardiovascular conditions presented to the emergency department 2 days after receiving his first dose of the Pfizer COVID-19 vaccine with symptoms of chest pain and dyspnea. The chest pain worsened with lying flat and improved with sitting upright. Cardiac examination was unremarkable and notable for the absence of a pericardial rub. A 12-lead ECG showed diffuse PR segment depression and PR segment elevation in lead aVR, consistent with acute pericarditis. On admission, the serum troponin I was 37 pg/ml (normal range 0.006–0.060 ng/ml) and serum C-reactive protein was 50 mg/dl (normal range 0–10 mg/l). SARS COVID-19 PCR testing, other viral serologies, and bacterial testing were all negative. Transthoracic echocardiography (TTE) showed a left ventricular (LV) ejection fraction (LVEF) of 55% and no pericardial effusion. Invasive coronary angiography was negative for obstructive coronary artery disease. CMR showed both subepicardial late gadolinium enhancement (LGE) and myocardial edema in the basal inferior, basal inferolateral, and apical lateral LV segments. Global longitudinal strain (GLS) by TTE and CMR was normal at − 19.0% and − 19.1% respectively (normal is < − 18%). The patient was diagnosed with acute myopericarditis, treated with high-dose aspirin and colchicine, and was discharged home in stable clinical condition after 48 h of observation.

#### Case 2

A 19-year-old male with history of asthma and without any known cardiovascular conditions presented to the emergency department one day after receiving his second dose of the Pfizer COVID-19 vaccine with symptoms of chest pain. The chest pain worsened with inspiration and was associated with dyspnea, nausea, and vomiting. Cardiac examination was unremarkable and notable for the absence of a pericardial rub. A 12-lead ECG showed sinus tachycardia without any ST-T abnormalities. On admission, the serum troponin I was elevated at 49 pg/ml and the serum C-reactive protein was elevated at 109 mg/l. SARS COVID-19 PCR testing, viral serologies, and bacterial testing were all negative. TTE showed an LVEF of 62% and no pericardial effusion. CMR showed subepicardial LGE in the basal inferolateral segment. T2-weighted images to assess myocardial edema were not obtained. GLS by TTE and CMR was reduced (−12.0% and −12.1% respectively). The patient was diagnosed with myopericarditis, treated with colchicine and high-dose ibuprofen, and was discharged home in stable clinical condition.

#### Case 3

A 25-year-old male with no known prior health problems presented to the emergency department 3 days after receiving his second dose of the Moderna COVID-19 vaccine with chest pain and dyspnea. A 12-lead ECG showed diffuse PR segment depression and PR segment elevation in lead aVR, consistent with acute pericarditis. On admission, the serum cardiac troponin was elevated at 17 pg/ml and the serum C-reactive protein was elevated at 96 mg/dl. SARS COVID-19 PCR testing, other viral serologies, and bacterial testing were all negative. TTE showed an LVEF of 60% and no pericardial effusion. The patient was diagnosed with acute myopericarditis, treated with colchicine, and was discharged home in stable clinical condition. CMR performed 1 week after hospital discharge showed subepicardial LGE and myocardial edema in the lateral LV segments. GLS by TTE and CMR was normal (< −18%).

#### Case 4

A 37-year-old male with no known health problems presented to the emergency department 2 days after receiving his second dose of the Pfizer COVID-19 vaccine with chest pain radiating to the left arm. A 12-lead ECG showed ST elevations in the lateral leads and ST depression in lead V1. He was taken emergently to the cardiac catheterization laboratory where invasive coronary angiography was negative for obstructive coronary artery disease. On admission, serum troponin I was elevated at 26 pg/ml and the erythrocyte sedimentation rate was 32 mm/h (normal range 0–15 mm/h). SARS COVID-19 PCR testing, other viral serologies, and bacterial testing were all negative. TTE showed an LVEF of 65% and no pericardial effusion. CMR showed subepicardial LGE in the basal anteroseptal segment. T2-weighted images showed no hyperintensity in any of the myocardial segments but T2 mapping showed elevated relaxation time, consistent with myocardial edema. GLS by TTE and CMR was borderline reduced at −18% and −16% respectively. The patient was diagnosed with acute myocarditis and was sent home without any medications with cardiology follow up.

#### Case 5

A 20-year-old male with no known health problems presented to the emergency department with chest pain and dyspnea 3 days after receiving his second dose of the Pfizer COVID-19 vaccine. A 12-lead ECG showed diffuse PR segment depression and PR segment elevation in lead aVR, consistent with acute pericarditis. On admission, serum troponin I was elevated at 58 pg/ml. SARS COVID-19 PCR testing, other viral serologies, and bacterial testing were all negative. TTE showed a borderline depressed LVEF of 51% and no pericardial effusion. CT coronary angiography showed no coronary artery calcification and non-obstructive coronary artery disease. CMR showed subepicardial and mid-myocardial LGE in the basal, mid, and apical lateral segments accompanied by myocardial edema in mid and apical lateral segments on T2-weighted images. GLS by TTE and CMR was reduced (−14% and −12% respectively). The patient was diagnosed with acute myopericarditis and was treated with colchicine, ibuprofen, lisinopril, and metoprolol tartrate. He was discharged home in stable clinical condition and was asymptomatic when seen in outpatient cardiology clinic follow-up 4 weeks after discharge.

## Discussion

Studies of mRNA-based COVID-19 vaccines have shown they are frequently associated with mild adverse effects in the first 24–48 h after administration, including pain and swelling at the injection site, fatigue, headache, muscle pain, chills, fever, and nausea [13]. While more severe adverse effects following COVID-19 vaccination are rare, they can occur.

In this case series, we provide detailed clinical and cardiovascular imaging findings in five young adult male patients with acute myocarditis following mRNA COVID-19 vaccination. With respect to the CMR findings in our patients, all five patients met the updated 2018 Lake Louise CMR criteria for acute myocarditis [14]. Interestingly, LGE and myocardial edema in our patients predominantly localized to the basal and mid lateral LV segments. Such distribution of myocardial edema and LGE on CMR was similarly seen in other case series of adults and pediatric population [5–10, 15, 16]. Prior studies suggest that this distribution of LGE carries a better prognosis compared to LGE localized to the septal segments [17].

Our case series adds to a growing number of recently published case series suggesting a possible association between acute myocarditis and mRNA COVID-19 vaccination, predominantly in males younger than 40 years of age. In the largest such series, by Montgomery et al., only 8 of the 23 patients (all males aged 20–51 years) underwent CMR [9]. Among these 8, all met Lake Louise CMR criteria for acute myocarditis but the specific pattern and location of LGE was not reported [9]. Similarly, Larson et al. described ‘patchy’ LGE in 8 healthy males aged 21 to 56 years diagnosed with acute myocarditis 2–4 days after receiving COVID-19 vaccination but again a more specific pattern was not described [8]. Other case series by Rosner et al., Shaw et al., Dickey et al. and Kim et al. found a pattern on CMR identical to the one we describe in our series [5–7, 10]. An additional case series by Marshall et al. found similar CMR findings in 7 adolescent male patients, suggesting that CMR findings in this clinical scenario may occur across age groups [11].

Consistent with previously published case series, all of our patients were males younger than 40 years. According to the CDC’s Advisory Committee on Immunization Practices, as of June 11, 2021, there have been 52 million doses of mRNA-based COVID-19 administered in persons aged 12–29 years and within this group there have been only 323 confirmed cases of myocarditis, pericarditis, or myopericarditis [18]. Among these 323 patients, the median age was 19 years, 291 (90%) were males, and the median interval from vaccination to symptom onset was 2 days [18]. From previous literature, it also appears that myocarditis typically occurs more commonly in males, and the incidence is highest amongst adolescent and young adults [19, 20]. The underlying pathophysiologic mechanism(s) of higher prevalence of acute myocarditis in males is poorly understood, but limited data suggest that elevated testosterone can contribute to acute myocarditis is by increasing viral binding to myocytes [21], inhibiting anti-inflammatory cell population [22], and upregulating cardiac fibrotic remodeling genes [23]. On the other hand, estrogen may play a protective role in myocarditis by stimulating inhibitory T regulator cells and inhibiting proinflammatory T-cells [23, 24].

If in fact there is a causal relationship between COVID-19 vaccination and acute myocarditis in this patient population—which this case series does not definitively establish—the underlying mechanism is unclear at this time. Rare cases of myocarditis have been reported after vaccinations for other diseases. Myocarditis and pericarditis have been reported after smallpox vaccination, and endomyocardial biopsy in such cases has suggested an immunological mediated etiology [25]. Because SARS-CoV-2 infection results a hyperinflammatory host response, it is plausible that vaccination stimulates in rare cases a similar immune response with direct cardiac effects.

Two-dimensional speckle tracking with echocardiography and feature-tracking with CMR can quantitatively measure myocardial mechanics (strain) in the longitudinal direction; and a decrease in GLS has been shown to detect LV dysfunction before a reduction in LVEF is identified [26]. Interestingly, three of our five patients had reduced or borderline reduced GLS. In acute myocarditis, reduced LV strain has shown to be predictive of adverse outcomes, even in patients with preserved LVEF [27]. Longer clinical follow-up may help understand if the CMR abnormalities observed are temporary in the setting of a high inflammatory state or longer-lasting, and whether they help predict any clinically-relevant adverse events or deterioration in cardiac function.

In the state of Rhode Island, a total of 693,578 people have received COVID-19 vaccination with at least one dose and a total of 635,432 people are fully vaccinated against COVID-19 infection [28]. This case series suggest that the estimated prevalence of acute myocarditis following COVID-19 vaccination is very low, but physicians should be vigilant in diagnosing such patients who are presenting with chest pain following COVID-19 vaccinations especially as we expand COVID-19 vaccination to more younger population. While, per CDC’s Advisory Committee on Immunization Practices, as of June 11, 2021, 96% of these patients were hospitalized, the vast majority had clinical courses similar to what we observed in all 5 of our patients—no deaths, resolution of symptoms with non-steroidal anti-inflammatory drugs, and relatively short hospital stays [18]. Nevertheless, continued clinical follow-up of these patients, perhaps with repeat CMR in 3–6 months, is needed to better understand whether there are any meaningful medium- and long-term effects of myocarditis following mRNA COVID-19 vaccination.

### Limitations

Given this is a small case series from a single tertiary center in the state of Rhode Island, it is important to highlight that we cannot provide causal relationship and in fact only provides a possible association of acute myocarditis with mRNA COVID-19 vaccination. We do not have a long term follow up data on these patients and unable to ascertain the relevance of acute myocarditis on potentially receiving a future booster dose of COVID-19 vaccination. Despite these limitations, it is notable that the clinical presentation and CMR findings in these patients appear consistent with previously published case series of acute myocarditis after receiving mRNA COVID-19 vaccination.

## Conclusion

In conjunction with other recently published case series and national vaccine safety surveillance data, this case series suggests an association between acute myocarditis and COVID-19 vaccination in young males and highlights a potential pattern in accompanying CMR abnormalities.

## Data Availability

These can be provided upon request to the corresponding author.

## Abbreviations

CDC: Centers of Disease Control and Prevention
CMR: Cardovascular magnetic resonance
COVID-19: Coronavirus disease of 2019
CT: Computed tomography
ECG: Electrocardiogram
FDA: United States Food and Drug Administration
GLS: Global longitudinal strain
LGE: Late gadolinium enhancement
LV: Left ventricle/left ventricular
LVEF: Left ventricular ejection fraction
mRNA: Messenger ribonucleic acid
SARS-CoV-2: Severe acute respiratory syndrome coronavirus 2
TTE: Transthoracic echocardiography
US: United States

## References

[1] WHO Coronavirus (COVID-19) Dashboard. https://covid19.who.int/. Accessed 31 May 2021.

[2] Interim Clinical Considerations for Use of COVID-19 Vaccines Currently Authorized in the United States. https://www.cdc.gov/vaccines/covid-19/clinical-considerations/covid-19-vaccines-us.html. Accessed 31 May 2021.

[3] COVID-19 Vaccinations in the United States. https://covid.cdc.gov/covid-data-tracker/#vaccinations. Accessed 31 May 2021.

[4] COVID-19 VaST Work Group Technical Report – May 17, 2021. https://www.cdc.gov/vaccines/acip/work-groups-vast/technical-report-2021-05-17.html. Accessed 31 May 2021.

[5] Shaw KE, Cavalcante JL, Han BK, Gossl M (2021). Possible association between COVID-19 vaccine and myocarditis; clinical and CMR findings. JACC.

[6] Dickey JB, Albert E, Badr M, Laraja KM, Sena LM, Gerson DS, Saucedo JE, Qureshi W, Aurigemma GO (2021). A series of patienets with myocarditis following SARS-CoV-2 vaccination with mRNA-1279 and BNT162b2. JACC.

[7] Kim HW, Jenista ER, Wendell DC et al. Patients with acute myocarditis following mRNA COVID-19 vaccination. JAMA Cardiol; 2021. 10.1001/jamacardio.2021.2828 PMC8243258 34185046

[8] Larson KF, Ammirati E, Adler ED et al. Myocarditis after BNT162b2 and mRNA-1273 vaccination. Circulation; 2021. 10.1161/CIRCULATIONAHA.121.055913 PMC8340725 34133884

[9] Montgomery J, Ryan M, Engler R et al. Myocarditis following immunization with mRNA COVID-19 vaccines in members of the US military. JAMA Cardiol; 2021. 10.1001/jamacardio.2021.2833 PMC8243257 34185045

[10] Rosner CM, Genovese L, Tehrani BN et al. Myocarditis temporally associated with COVID-19 vaccination. Circulation; 2021. 10.1161/CIRCULATIONAHA.121.055891 PMC8340723 34133885

[11] Marshall M, Ferguson ID, Lewis P et al. Symptomatic acute myocarditis in seven adolescents following Pfizer-BioNTech COVID-19 vaccination. Pediatrics; 2021. 10.1542/peds.2021-052478 34088762

[12] Clinical Considerations: Myocarditis and Pericarditis after Receipt of mRNA COVID-19 Vaccines Among Adolescents and Young Adults. https://www.cdc.gov/vaccines/covid-19/clinical-considerations/myocarditis.html. Accessed May 31 2021.

[13] Possible Side Effects After Getting a COVID-19 Vaccine. https://www.cdc.gov/coronavirus/2019-ncov/vaccines/expect/after.html. Accessed May 31 2021.

[14] Ferreira VM, Schulz-Menger J, Holmvang G (2018). Cardiovascular magnetic resonance in nonischemic myocardial inflammation: expert recommendations. J Am Coll Cardiol.

[15] Mouch SA, Roguin A, Hellou E, Ishai A, Shoshan U, Mahamid L, Zoabi M, Aisman M, Goldshmidt N, Yanay NB. Myocarditis following COVID-19 mRNA vaccination. Vaccine; 2021. 10.1016/j.vaccine.2021.05.087 PMC8162819 34092429

[16] Marshall M, Ferguson ID, Lewis P, Jaggi P, Gagliardo C, Collins JS, Shaughnessya R, Carona R, Fuss C, Corbin KJ, Emuren L (2021). Symptomatic acute myocarditis in seven adolescents following Pfizer-BioNTech COVID-19 vaccination. Pediatrics.

[17] Aquaro GD, Perfetti M, Camastra G (2017). Cardiac MR with late gadolinium enhancement in acute myocarditis with preserved systolic function: ITAMY study. J Am Coll Cardiol.

[18] Centers for Disease Control and Prevention. Morbidity and Mortality Weekly Reports. Use of mRNA COVID-19 Vaccine After Reports of Myocarditis Among Vaccine Recipients: Update from the Advisory Committee on Immunization Practices — United States, June 2021. Accessed July 7, 2021. 10.15585/mmwr.mm7027e2 PMC8312754 34237049

[19] Kyto V, Sipila J, Rautava P (2013). The effects of gender and age on occurrence of clinically suspected myocarditis in adulthood. Heart.

[20] Vasudeva R, Bhatt P, Lilje C (2021). Trends in acute myocarditis related pediatric hospitalizations in the United States, 2007–2016. Am J Cardiol.

[21] Lyden DC, Olszewski J, Feran M, Job LP, Huber SA (1987). Coxsackievirus B-3-induced myocarditis. Effect of sex steroids on viremia and infectivity of cardiocytes. Am J Pathol.

[22] Frisancho-Kiss S, Coronado MJ, Frisancho JA (2009). Gonadectomy of male BALB/c mice increases Tim-3(+) alternatively activated M2 macrophages, Tim-3(+) T cells, Th2 cells and Treg in the heart during acute coxsackievirus-induced myocarditis. Brain Behav Immun.

[23] Fairweather D, Cooper LT, Blauwet LA (2013). Sex and gender differences in myocarditis and dilated cardiomyopathy. Curr Probl Cardiol.

[24] Li Z, Yue Y, Xiong S (2013). Distinct Th17 inductions contribute to the gender bias in CVB3-induced myocarditis. Cardiovasc Pathol.

[25] Eckart RE, Love SS, Atwood JE (2004). Incidence and follow-up of inflammatory cardiac complications after smallpox vaccination. J Am Coll Cardiol.

[26] Mondillo S, Galderisi M, Mele D (2011). Speckle-tracking echocardiography: a new technique for assessing myocardial function. J Ultrasound Med.

[27] Hsiao JF, Koshino Y, Bonnichsen CR (2013). Speckle tracking echocardiography in acute myocarditis. Int J Cardiovasc Imaging.

[28] Rhode Island COVID-19 Vaccine Tracker. https://data.rgj.com/covid-19-vaccine-tracker/rhode-island/44/. Accessed July 12, 2021.

